# Inhibition of USP2 suppresses pathological pexophagy and rescues PEX1-deficient peroxisome biogenesis disorder

**DOI:** 10.1038/s41392-026-02882-3

**Published:** 2026-09-24

**Authors:** Yong Hwan Kim, Doo Sin Jo, Ae-Kyeong Kim, Kyu Sun Lee, Dong-Hyung Cho

**Affiliations:** 1 School of Life Sciences, BK21 FOUR KNU Creative BioResearch Group, Kyungpook National University, Daegu, Republic of Korea; 2 ORGASIS Corp., Suwon, Republic of Korea; 3 Metabolism and Neurophysiology Research Group, KRIBB, Daejeon, Republic of Korea

**Keywords:** Cell biology, Molecular biology

**Dear Editor**,

Peroxisome biogenesis disorders (PBDs) are ultrarare, life-limiting inherited diseases in which functional peroxisomes are pathologically lost, driving progressive multisystem failure dominated by neurological involvement.^1^ Clinically, PBDs are encompassed within the Zellweger spectrum disorders (ZSDs), which range from life-threatening neonatal-onset disease to milder forms with lifelong neurodevelopmental and motor impairments.^1^ Genetically, ZSD is caused by biallelic variants in *Peroxin* (*PEX*) genes, with *PEX1* being the predominant locus, accounting for ~65% of cases across cohorts.^1^ PEX1 belongs to the peroxisomal AAA-ATPase complex required for matrix-protein import; therefore, PEX1 defects primarily impair peroxisomal enzyme import. Recent work further indicates that PEX1 mutation or loss can provoke excessive peroxisome-selective autophagy (pathological pexophagy), accelerating peroxisome depletion; although broad autophagy blockade (e.g., chloroquine/CQ) provides proof-of-concept rescue, it is inherently nonselective and poses mechanistic and translational liabilities.^2^ Because peroxisome abundance reflects the balance between organelle biogenesis/import and selective turnover, PEX1-mutant cells may lose peroxisomes not only through defective matrix-protein import but also through active removal of damaged peroxisomes. Because pexophagy is initiated by ubiquitination of peroxisomal membrane proteins, upstream ubiquitin-editing machinery may provide a more selective target than global autophagy blockade.^2–4^ We therefore hypothesized that defective PEX1 drives pathological pexophagy through a druggable ubiquitination-linked regulator, whose targeting could selectively modulate maladaptive pexophagy.

To identify a druggable checkpoint for PEX1-defective contexts, we screened a focused ubiquitin-related compound library in PEX1-G843D patient-derived fibroblasts using ABCD3-positive peroxisome puncta as the primary readout. ML364, a ubiquitin-specific peptidase 2 (USP2) inhibitor, was selected as a top candidate that restored peroxisome abundance (Supplementary Fig. 1a). Consistent with the pharmacological results, USP2 depletion increased peroxisome abundance in PEX1-G843D fibroblasts (Fig. 1a, left). In parallel, ML364 dose-dependently restored ABCD3-positive peroxisome puncta in PEX1-G843D fibroblasts without affecting peroxisome abundance in wild-type fibroblasts and similarly rescued peroxisome abundance in an independent PEX1-mutant patient-derived fibroblast line, GM16513 carrying PEX1 2097insT (Supplementary Fig. 1b–d). Notably, lipid analysis further showed that ML364 reduced the elevated C26:0 lysoPC level, C24:0 lysoPC level and C26:0/C16:0 ratio in PEX1-G843D fibroblasts (Fig. 1a, right). Collectively, these results indicate that pharmacological inhibition of USP2 significantly restores peroxisome abundance and partially ameliorates VLCFA-related abnormalities in PEX1-defective patient-derived cells.

**Fig. 1:**
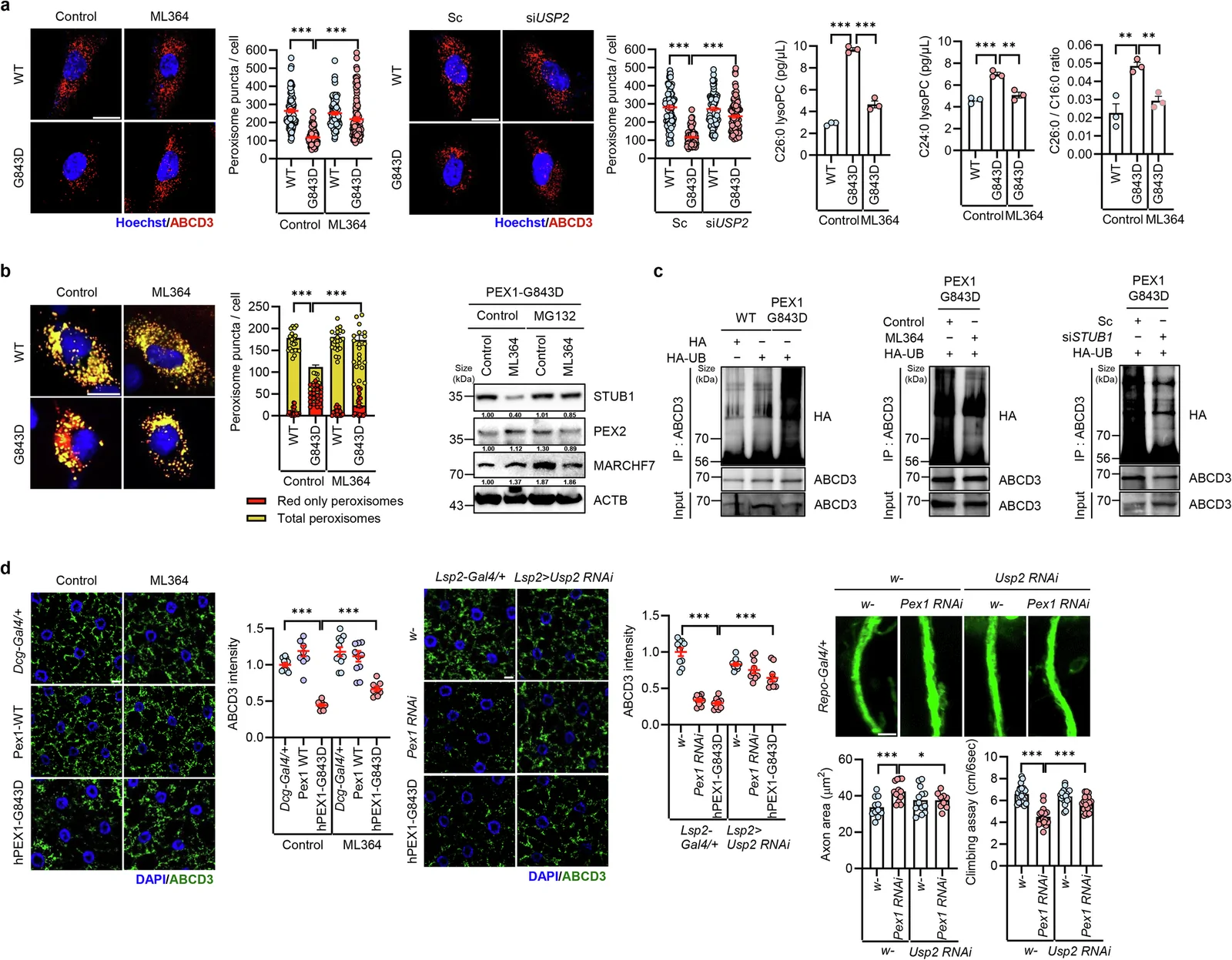
USP2 perturbation suppresses pathological pexophagy and peroxisome loss in PEX1-defective ZSD models. **a** Wild-type (WT) or PEX1-G843D fibroblasts (G843D) were treated with ML364 (10 µM) for 48 h, immunostained with an anti-ABCD3 antibody, labeled with Hoechst 33342 to visualize nuclei, and imaged by confocal microscopy; peroxisome abundance was quantified as the number of ABCD3-positive puncta per cell (≥100 cells) (left). WT or PEX1-G843D fibroblasts were transfected with scrambled siRNA (Sc) or siRNA targeting *USP2* (si*USP2*) for 72 h, followed by immunostaining with an anti-ABCD3 antibody and staining with Hoechst 33342 to label nuclei; the number of peroxisomes per cell was quantified (≥100 cells) (middle) (scale bar, 20 µm). WT fibroblasts or PEX1-G843D fibroblasts were treated with ML364 (10 µM) for 48 h, followed by tip sonication and MTBE-based lipid extraction and LC–MS-based quantification of VLCFA-related readouts, including the C26:0/C16:0 ratio, C26:0 lysoPC, and C24:0 lysoPC. (*n* = 3) (right). **b** For pexophagic flux analysis, wild-type (WT) or PEX1-G843D fibroblasts (G843D) were transfected with EGFP–mCherry–PEX26TM and treated with ML364 (10 µM) for 48 h. Cells were stained with Hoechst 33342 to label nuclei, and confocal images were acquired. Peroxisomes were classified as EGFP(+)/mCherry(+) dual-positive peroxisomes (total peroxisomes, yellow) or EGFP(−)/mCherry(+) red-only puncta (red-only peroxisomes, red), representing cytosolic peroxisomes and lysosome-delivered peroxisomes, respectively. The number of red-only and total peroxisomes per cell were quantified (scale bar, 20 µm) (left). For biochemical analyses in PEX1-G843D fibroblasts, cells were treated with ML364 (10 µM) for 48 h and/or MG132 (10 µM, 12 h) as indicated, and lysates were analyzed by immunoblotting with antibodies against STUB1, PEX2, MARCHF7, and ACTB (right). **c** To assess ABCD3 ubiquitination, WT or PEX1-G843D fibroblasts were transfected with HA-tagged ubiquitin (HA-UB), followed by immunoprecipitation using an anti-ABCD3 antibody and immunoblotting with the indicated antibodies (left). PEX1-G843D fibroblasts transfected with HA-UB were further treated with ML364 (middle) or transfected with si*STUB1* (right), as indicated, followed by anti-ABCD3 immunoprecipitation and immunoblotting to assess ABCD3 ubiquitination. **d** Fat bodies from fat body–specific PEX1 wild-type–expressing (*Dcg* > *PEX1-WT*, PEX1-WT) or hPEX1-G843D–expressing flies (*Dcg* > *hPEX1-G843D*, hPEX1-G843D), treated with ML364 (50 µM), together with control flies (*Dcg-Gal4/+*), were immunostained with an anti-ABCD3 antibody, and the fluorescence intensity of ABCD3-positive puncta was quantified (left). Fat bodies from fat body–specific *Pex1* knockdown or hPEX1-G843D–expressing flies with or without *Usp2* knockdown (*Lsp2>Usp2 RNAi*), together with control flies (*Lsp2-Gal4/+*), were immunostained with an anti-ABCD3 antibody, and the fluorescence intensity of ABCD3-positive puncta was quantified (middle). The A3 abdominal ventral longitudinal muscles (VLM) of control flies (*Repo-Gal4/+, W-*) and flies with glia-specific *Pex1* (*Repo>Pex1 RNAi, Pex1 RNAi*) and *Usp2* (*Repo>Usp2 RNAi, Usp2 RNAi*) knockdown were immunostained with an anti-HRP antibody, and the axonal area was quantified (scale bar, 0.5 µm). Thirty-day-old control flies (*W-)* and flies with glia-specific *Pex1* (*Repo>Pex1 RNAi, Pex1 RNAi*) and *Usp2* (*Repo>Usp2 RNAi, Usp2 RNAi*) knockdown were assessed for climbing ability (*n* ≥ 10) (right). (Data are presented as the mean ± SEM. Statistical significance was determined by one-way ANOVA followed by post hoc test; *p < 0.05, **p < 0.01, ***p < 0.001)

To assess pathological pexophagy under defective PEX1 conditions, we used the EGFP–mCherry–PEX26_TM_ tandem reporter, which monitors peroxisome delivery to lysosomes without relying on PTS1-dependent matrix import. Upon lysosomal delivery, EGFP fluorescence is easily quenched while mCherry is retained, producing EGFP(−)/mCherry(+) “red-only” puncta as a readout of pexophagic flux. PEX1-G843D fibroblasts exhibited markedly increased red-only puncta compared with wild-type cells, and ML364 reduced these puncta while preserving EGFP(+)/mCherry(+) dual-positive peroxisomes, consistent with suppressed pexophagic flux and restored peroxisome abundance (Fig. 1b, left). Mechanistically, among the validated pexophagy-related E3 ligases examined in PEX1-G843D fibroblasts, ML364 selectively reduced STUB1 but not PEX2 or MARCHF7. The ML364-induced reduction in STUB1 expression was reversed by proteasome inhibition, indicating that USP2 inhibition promotes proteasomal STUB1 destabilization and supports STUB1 as the most relevant downstream E3 ligase in PEX1-G843D fibroblasts (Fig. 1b, right). In parallel, defective PEX1 increased ABCD3 ubiquitination, whereas ML364 markedly suppressed ABCD3 ubiquitination in PEX1-G843D fibroblasts (Fig. 1c, left). Moreover, coimmunoprecipitation analysis showed that USP2 interacts with STUB1 and that USP2 depletion increased STUB1 polyubiquitination in PEX1-G843D fibroblasts (Supplementary Fig. 1f, g). Consistently, STUB1 depletion suppressed ABCD3 ubiquitination in PEX1-G843D fibroblasts, further linking STUB1 to USP2-dependent regulation of peroxisomal membrane-protein ubiquitination (Fig. 1c, right). STUB1 depletion also rescued PEX1 depletion-induced peroxisome loss (Supplementary Fig. 1e). Collectively, these data support a model in which USP2 regulates STUB1 stability and thereby contributes to pathological pexophagy under PEX1-defective conditions.

To establish in vivo relevance, we used *Drosophila melanogaster* models of PEX1 deficiency and a humanized PEX1-G843D PBD model. Fat body–specific Pex1 depletion or fat body expression of hPEX1-G843D caused loss of peroxisomes (Fig. 1d, left). Remarkably, pharmacological inhibition of USP2 with ML364 restored peroxisome abundance in the humanized PEX1-G843D in vivo model associated with pexophagy (Fig. 1d, left). Moreover, tissue-specific Usp2 depletion produced a comparable rescue, thereby genetically validating Usp2 as the relevant on-target mediator in vivo (Fig. 1d, middle). Together, these results demonstrate that genetic or pharmacological targeting of Usp2 reverses PEX1 perturbation-driven peroxisome depletion in vivo, supporting USP2 as a druggable therapeutic target for suppressing pathological pexophagy. It was reported that clinical studies and *Drosophila melanogaster* models link ZSD/peroxisome deficiency to axonal swelling with accompanying motor impairment.^5^ In 30-day-old flies, glial Pex1 depletion-induced axonal swelling at neuromuscular junctions in the abdominal ventral longitudinal muscle and significantly reduced climbing performance (Fig. 1d, right). Notably, glial depletion of Usp2 attenuated axonal swelling and improved locomotor activity in the Pex1-depleted context (Fig. 1d, right). Collectively, these findings support USP2 as a targetable determinant of defective PEX1-associated neuro-axonal pathology and motor dysfunction in vivo.

In conclusion, we identified USP2 as a regulator of pathological pexophagy in PEX1-defective contexts. Across PEX1-defective models—from patient-mutant fibroblasts to *Drosophila melanogaster*—USP2 suppression restored peroxisome abundance and mitigated disease-associated phenotypes. Mechanistically, USP2 maintained STUB1 stability, promoting the ubiquitination of peroxisomal membrane proteins and sustaining aberrant pexophagy signaling under PEX1 depletion. These findings highlight USP2 as an actionable target for PEX1-associated PBD-ZSDs by selectively modulating maladaptive pexophagy rather than broadly inhibiting core autophagy.

These findings should be interpreted in the context that defective matrix protein import remains the primary pathogenic defect in PBD-ZSD, whereas pathological pexophagy may further contribute to peroxisome loss in PEX1-associated models. Unlike nonselective late-stage autophagy inhibitors such as CQ, which block autophagic flux after autophagosome closure, USP2 inhibition appears to act earlier by suppressing the initiation step of ubiquitin-dependent pexophagy.^2,4^ This upstream intervention may preserve residual, partially functional peroxisomes. Future studies should define the USP2–STUB1 interaction and the ubiquitination site(s) driving STUB1 destabilization. In addition, optimization of USP2 inhibitors will require ADME and pharmacological profiling and validation in higher-order PBD models, including iPSC-derived disease and mouse models, as well as additional PEX-associated ZSD contexts beyond PEX1.

Collectively, our findings suggest a mechanistic framework for developing pexophagy-directed interventions aimed at system-level correction of defective PEX1-associated cellular dysfunction rather than symptomatic support.

## Supplementary information

Supplemental Material

## Data Availability

All data supporting the findings of this study are available within the article and its supplementary materials. Additional supplementary data supporting this study have been deposited in Figshare under the reserved https://doi.org/10.6084/m9.figshare.32657454. Source data for the main figure are available from Dong-Hyung Cho (dhcho@knu.ac.kr) and Kyu Sun Lee (ekuse74@kribb.re.kr).
