# Supplementary material for: Inhibition of USP2 suppresses pathological pexophagy and rescues PEX1-deficient peroxisome biogenesis disorder
Source: Signal Transduct Target Ther. 2026 Sep 24;11:405. doi: 10.1038/s41392-026-02882-3 (PMC13601545; doi:10.1038/s41392-026-02882-3)
Supplement: Supplementary file 1 [file 41392_2026_2882_MOESM1_ESM.docx]

**Supplementary Materials for**

**Inhibition of USP2 suppresses pathological pexophagy and rescues PEX1-deficient peroxisome biogenesis disorder**

Yong Hwan Kim^1#^, Doo Sin Jo^2#^, Ae-Kyeong Kim^3^, Kyu Sun Lee^3*^, and Dong-Hyung Cho^1*^

^1^School of Life Sciences, BK21 FOUR KNU Creative BioResearch Group, Kyungpook National University, Daegu 41566, Republic of Korea; ^2^ORGASIS, Suwon 08826, Republic of Korea; ^3^Metabolism and Neurophysiology Research Group, KRIBB, Daejeon 34141, Republic of Korea

**Materials and Methods**

***Reagents, siRNAs, and Plasmids***

A ubiquitination compound library for drug screening was obtained from TargetMol (Boston, MA, USA). ML364 and Bafilomycin A_1_ were obtained from Sigma-Aldrich (St. Louis, MO, USA). MG132 was obtained from Cayman Chemical Co (Ann Arbor, MI, USA). Validated short interfering RNAs (siRNAs) targeting *PEX1* (5'-GGACAAGAUUGGUGGGUUAUU-3'), *USP2* (5'-CAGAUUGUGGUUACUGUUCUAUU-3'), *STUB1* (5'-CUUCGAAUCGCGAAGAAGAUU-3'), and scrambled siRNA (5'-CCUACGCCACCAAUUUCGU-3') were synthesized by Bioneer Corporation (Daejeon, Korea). The HA-tagged pcDNA3.1 plasmid was obtained from Addgene (deposited by Oskar Laur). The HA-tagged ubiquitin WT (HA-UB) plasmid was obtained from Addgene (deposited by Ted Dawson). The Myc-STUB1 WT plasmid kindly proved by Dr. M. J. Lee (Seoul National University, Korea). pEGFP–mCherry–PEX26_TM_ kindly provided by Dr. P. K. Kim (University of Toronto, Toronto, ON, Canada)

***Cell Culture and Establishment of Stable Cell Lines***

HeLa cells were obtained from the American Type Culture Collection (CCL-2). PEX1-G843D fibroblasts were kindly provided by Dr. P. K. Kim (University of Toronto). Human fibroblasts (GM16513) from a ZSD patient with a *PEX1* 1-bp insertion (*c.2097-2098insT*) and healthy control fibroblasts (GM05756) were obtained from the Coriell Institute for Medical Research (Camden, NJ, USA). HeLa cells were cultured at 37°C in a 5% CO₂ incubator and cultured in DMEM (WELGENE; Korea) supplemented with 10% FBS (Hyclone; Logan, UT, USA) and 1% penicillin-streptomycin (WELGENE). Fibroblasts were cultured in DMEM supplemented with 15% FBS and 1% penicillin-streptomycin under the same incubation conditions.

***Western Blotting and Immunoprecipitation***

Cell lysates were prepared using 2× Laemmli sample buffer (Bio-Rad; Hercules, CA, USA). Total protein concentration was measured using Bradford dye (Bio-Rad), following the manufacturer’s instructions. Samples were separated by SDS-polyacrylamide gel electrophoresis (SDS-PAGE) and transferred onto a PVDF membrane (Bio-Rad). After blocking with 4% skim milk (BD Bioscience, San Jose, CA, USA) in TBST buffer, membranes were incubated with primary antibodies, including: anti-ABCD3 (1:3000) antibody obtained from Abcam (Cambridge, UK); anti-HA (1:1000) and anti-MARCHF7 (1:1000) antibodies were purchased from Santa Cruz Biotechnology (Dallas, TX, USA); anti-STUB1 (1:3000) antibody purchased from Bethyl laboratories (Montgomery, TX, USA); anti-ACTB (1:10000) antibody was purchased from Proteintech (Rosemont, IL, USA); anti-PEX2 (1:1000) was purchased from Invitrogen (Carlsbad, CA, USA). For detection, membranes were incubated with HRP-conjugated secondary antibodies were purchased from Cell Signaling Technology (Danvers, MA, USA)

Cells were lysed for 1 h at 4°C in RIPA buffer (iNtRON Biotechnology; Korea) with protease inhibitors (GenDEPOT). The supernatant was incubated with antibodies against USP2 (Abcepta, Inc.; San Diego, CA, USA), Myc (Santa Cruz Biotechnology), and ABCD3 (Santa Cruz Biotechnology) for 24 h at 4°C. Protein A/G PLUS-agarose (Santa Cruz Biotechnology) was then added, and samples were incubated for 2–4 h at 4°C. Immunoprecipitated proteins were analyzed by western blotting.

***Confocal Microscopy and Determination of Pexophagic Cells***

To quantify pexophagy, the cells were cultured on glass coverslips, treated with chemicals, or transfected with siRNA for various durations. The cells were then washed with PBS (WELGENE), fixed with 4% paraformaldehyde (Biosesang; Korea) for 20 min, and stained with the indicated dyes or antibodies. Nuclei were stained with Hoechst 33342 (Thermo Fisher Scientific, Waltham, MA, USA). Punctate peroxisomal structures were visualized using a confocal microscope (Leica Microsystems; Wetzlar, Germany). The number of peroxisome puncta was quantified with ABCD3 protein using ImageJ Fiji. Experiments were conducted at least three times for each condition, as indicated in each figure. The peroxisome count was determined by dividing the number of peroxisomal structures by the cell volume.

***LC-MS/MS Analysis of LysoPC Species and LC-MS-Based Determination of the C26:0/C16:0 Fatty Acid Ratio***

LysoPC 24:0 and LysoPC 26:0 were quantified from cell pellets by LC-MS/MS. The C26:0/C16:0 fatty acid ratio was measured by LC-MS using PBS-suspended cell samples containing ~1 × 10⁶ cells per sample. Lipids were extracted using an MTBE-based protocol. Samples were sonicated with a Qsonica Q55 tip sonicator (Qsonica, LLC; Newtown, CT, USA) at amplitude 15–20 for 1 min, mixed with 300 μL methanol and 1 mL MTBE, vortexed for 1 h, mixed with 150 μL water, vortexed for 5 min, and centrifuged at 3,000 × g for 10 min. The upper organic phase was collected, and the residual phase was re-extracted with 300 μL MTBE. Combined organic phases were dried in a SpeedVac concentrator (Thermo Fisher Scientific) and reconstituted in 100 μL methanol:chloroform, 9:1, v/v. Five microliters of each extract were injected. LysoPC analysis was performed on a Shimadzu Nexera LC system (Shimadzu Corporation; Japan) coupled to an AB Sciex QTRAP 6500+ mass spectrometer (SCIEX; Framingham, MA, USA) using an ACQUITY BEH C18 column, 2.1 × 150 mm, 1.7 μm, at 40°C. Mobile phase A was water:acetonitrile, 8:2, v/v, with 10 mM ammonium formate; mobile phase B was methanol:acetonitrile:isopropanol, 2:2:6, v/v/v, with 10 mM ammonium formate. The flow rate was 200 μL/min. The gradient was 60% B from 0–1 min, 80% B at 5 min, 98% B from 12–25 min, and 60% B from 26–30 min. LysoPC 24:0 and LysoPC 26:0 were detected by MRM using m/z 608.3 → 184.1 and 636.3 → 184.1, respectively. Quantification used external calibration curves from 0.78–25 pg/μL. For fatty acid ratio analysis, LC-MS was performed on an Agilent 1290 Infinity II LC system coupled to an AB Sciex Q-TOF 5600+ mass spectrometer using an InfinityLab Poroshell 120 EC-C18 column, 2.1 × 50 mm, 1.9 μm, at 50°C. The flow rate was 400 μL/min. The gradient was 40% B from 0–1 min, 60% B at 4 min, 98% B from 20–25 min, and 40% B from 26–30 min. Data were acquired in negative ion mode over m/z 150–1200. The C26:0/C16:0 ratio was calculated from MS1 extracted ion chromatogram peak areas.

***Drosophila melanogaster culture and Immunohistochemistry***

All fly stocks were maintained on standard cornmeal medium at 25℃ and 50% humidity under a 12h light/12h dark cycle. Fly stocks of *UAS-Pex1 RNAi* (BL#51497), *Lsp2-GAL4* (BL#6357), *Repo-GAL4* (BL#7415) and *Actin5C-GAL4* (BL#4414) were obtained from the Bloomington Stock Center (Bloomington, IN, USA). *UAS-Usp2 RNAi* (VDRC#104382) fly line was obtained from the Vienna Drosophila Resource Center (Vienna, Austria). *Dcg-GAL4* and was gift from Dr. J. Graff (UT Southwestern, TX, USA). *UAS-Pex1-WT* and *UAS-hPex1-G843D* fly lines were kindly provided by Prof. Andrew Simmonds. For ML364 (Sigma-Aldrich) treatment, flies were raised on food containing ML364 (50 µM) until the third-instar larval stage.

Third-instar larval fat bodies or adult female abdomens were dissected in phosphate-buffered saline (PBS) and subsequently fixed in 4% formaldehyde (Sigma-Aldrich). Following fixation, tissues were incubated in a blocking solution containing 4% bovine serum albumin (GenDEPOT) for 30 min. Samples were then incubated overnight at 4 °C with an anti-ABCD3 antibody. After washing, tissues were treated with an Alexa Fluor™ 488–conjugated secondary antibody (Thermo Fisher Scientific) for 2 h at room temperature in the dark. The samples were washed four times with PBST at 20 min intervals and mounted using Antifade Mounting Medium containing DAPI (Vector Laboratories; Newark, CA, USA). Fluorescence images were acquired using an Olympus FluoView confocal microscope (Olympus; Japan). Quantification of ABCD3-positive peroxisomal signal intensity was performed using ImageJ software, and statistical analyses were conducted with GraphPad Prism 10.

***RNA preparation and quantitative real-time PCR***

Adult male flies aged 5~7 days were collected, and total RNA was extracted using the easy-BLUE™ Total RNA Extraction Kit (iNtRON Biotechnology). And then we performed cDNA synthesis using SuperScript IV Reverse Transcriptase (Invitrogen; Carlsbad, CA, USA). Quantitatibe real-time PCR analysis was conducted using a StepOnePlus (Applied Biosystems; Foster City, CA, USA) with Power SYBRTM Green PCR Master Mix. The primers used for this study were Pex1(F)- ACGTAGATCCAAACACCCC, Pex1(R)- GACTTTTTCAGCGACTCCC, USP2(F)- GTACAGGAACAGCAGCGATG, USP2(R)- GGTACCTCCTCCGCTTGATT, rp49(F)-AGGGTATCGACAACAGAGTG and rp49(R)-CACCAGGAACTTCTTGAATC. Relative mRNA expression levels were calculated as fold changes and normalized to rp49 mRNA.

***Climbing assay***

Locomotor ability was assessed using a negative geotaxis (climbing) assay. Adult female flies aged 30 days were individually placed into transparent vials. Flies were gently tapped to the bottom of the vial to initiate the assay, and the distance climbed vertically along the vial wall was measured 6 s after tapping. The climbing distance was recorded in centimeters for each individual fly and used for subsequent quantitative analysis*.*

***Statistical Analysis***

Colocalization analysis was performed using ImageJ Fiji and Pearson’s correlation coefficient. Data were collected from at least three independent experiments and presented as mean ± standard error of the mean (SEM). Statistical analysis was conducted using one-way ANOVA, with **p* < 0.05, ***p* < 0.01, ****p* < 0.001 considered significant.
